# Transmission dynamics of COVID-19 in household and community settings in the United Kingdom, January to March 2020

**DOI:** 10.2807/1560-7917.ES.2022.27.15.2001551

**Published:** 2022-04-14

**Authors:** Jamie Lopez Bernal, Nikolaos Panagiotopoulos, Chloe Byers, Tatiana Garcia Vilaplana, Nicki Boddington, Xu-Sheng Zhang, Andre Charlett, Suzanne Elgohari, Laura Coughlan, Rosie Whillock, Sophie Logan, Hikaru Bolt, Mary Sinnathamby, Louise Letley, Pauline MacDonald, Roberto Vivancos, Obaghe Edeghere, Charlotte Anderson, Karthik Paranthaman, Simon Cottrell, Jim McMenamin, Maria Zambon, Gavin Dabrera, Mary Ramsay, Vanessa Saliba

**Affiliations:** 1 Immunisation and Countermeasures Department, Public Health England, London, United Kingdom; 2 Statistics, Modelling and Economics Department, Public Health England, London, United Kingdom; 3 Field Services Division, Public Health England, London, United Kingdom; 4 Public Health Wales, Cardiff, United Kingdom; 5 Health Protection Scotland, Glasgow, United Kingdom; 6 Microbiology Operations, Public Health England, London, United Kingdom; 7 TARGET Department, Public Health England, London, United Kingdom

**Keywords:** COVID-19, Transmission dynamics, Secondary Attack Rate, serial interval, Incubation period, Reproduction number

## Abstract

**Background:**

Households appear to be the highest risk setting for COVID-19 transmission. Large household transmission studies in the early stages of the pandemic in Asia reported secondary attack rates ranging from 5 to 30%.

**Aim:**

We aimed to investigate the transmission dynamics of COVID-19 in household and community settings in the UK.

**Methods:**

A prospective case-ascertained study design based on the World Health Organization FFX protocol was undertaken in the UK following the detection of the first case in late January 2020. Household contacts of cases were followed using enhanced surveillance forms to establish whether they developed symptoms of COVID-19, became confirmed cases and their outcomes. We estimated household secondary attack rates (SAR), serial intervals and individual and household basic reproduction numbers. The incubation period was estimated using known point source exposures that resulted in secondary cases.

**Results:**

We included 233 households with two or more people with 472 contacts. The overall household SAR was 37% (95% CI: 31–43%) with a mean serial interval of 4.67 days, an R_0_ of 1.85 and a household reproduction number of 2.33. SAR were lower in larger households and highest when the primary case was younger than 18 years. We estimated a mean incubation period of around 4.5 days.

**Conclusions:**

Rates of COVID-19 household transmission were high in the UK for ages above and under 18 years, emphasising the need for preventative measures in this setting. This study highlights the importance of the FFX protocol in providing early insights on transmission dynamics.

## Introduction

As of the end of July 2020, more than 17 million cases of coronavirus disease (COVID-19) had been reported globally with more than 660,000 deaths [1]. The causative agent, severe acute respiratory syndrome coronavirus 2 (SARS-CoV-2), is primarily transmitted through the droplet route although aerosol, contact and faecal transmission may also contribute [2,3].

Investigations of household transmission dynamics were reported early in the pandemic in China and other countries in Asia [4-12]. Households appeared to be the highest risk setting for transmission with reported symptomatic secondary attack rates (SAR) in household contacts ranging from 5% to 30% [4-10]. Rates of symptomatic infection increase with age and risk factors for more severe disease include age, male sex and a range of comorbidities [13,14]. As countries move from broad physical distancing measures to more targeted approaches, a detailed understanding of risk factors for transmission is increasingly important.

In the United Kingdom (UK), the first cases of COVID-19 were reported in late January 2020, the number of cases rapidly increased from March before plateauing, then declining after physical distancing measures were introduced [13]. We followed up the first few hundred (FF100) cases of COVID-19 in the UK and their household contacts using the World Health Organization (WHO) 'first few X cases and contacts' (FFX) protocol that was established before the COVID-19 pandemic [15]. We have previously reported on the characteristics and outcomes of these cases [13]. Here we describe the transmission dynamics and risk factors for transmission and acquisition of symptomatic infection.

## Methods

### Study design

We used a prospective case-ascertained study design based on the WHO FFX protocol [15].

### Ascertainment of cases and contacts

The case ascertainment has been described in detail elsewhere [13]. Briefly, in the early stages of the pandemic (January and early February 2020 with primarily imported cases and their contacts) all PCR-positive cases who met the case definition were followed up using enhanced surveillance forms upon identification and after 14 days. This was later (from March 2020) restricted to indigenous cases only. We recruited cases from January to March 2020.

Close contacts of confirmed cases were identified by the local Health Protection Team (HPT). Those considered at greatest risk, including household contacts, others with direct face-to-face contact and healthcare workers who had not worn recommended personal protective equipment were actively followed up on a daily basis for 14 days and asked about relevant symptoms. Household contacts were defined as those living or spending substantial time (overnight) in the same household. Other contacts not classified as close contacts were provided with health advice and advised to contact the HPT if they developed relevant symptoms. Guidance at the time was that contacts should be PCR-tested if and when they developed symptoms. The HPTs completed enhanced surveillance questionnaires to collect details from cases on symptoms, medical history, details of the exposure, outcome and any virological tests (in Supplement 1 contains the initial contact questionnaire). A team of trained staff (health protection practitioners, nurses, doctors and field epidemiologists) proactively followed up all household contacts of confirmed cases 14 days or more after symptom onset in the index case using telephone interviews to assess subsequent development of any symptoms and final outcomes (Supplement 2 contains the follow-up questionnaire). Contacts who developed symptoms compatible with COVID-19 were offered PCR testing as per national guidance [13].

If cases or contacts could not be contacted by telephone after at least two attempts, or if health protection teams had recorded a request for no further contact, they were classified as lost to follow-up.

Details on other non-household community contacts were obtained through the HPZone public health management system. Community contacts with any point source exposures were included where there were no other suspected exposures and complete information was available on the timing of the exposure and symptom onset in the contact. Healthcare workers, returning travellers and airplane exposures were excluded. We also maintained a detailed dataset with information on community exposures and outcomes among all possible contacts of the first six cases. Full tracing of community exposures was stopped when community transmission was established and separate contact tracing systems were established.

### Analysis

#### Household analysis

Confirmed cases were those who tested positive for SARS-CoV-2 by PCR. Probable cases were those with fever (≥ 38 °C, self-reported), anosmia or respiratory symptoms. Those who had other unrelated or pre-existing illnesses were excluded. FF100 cases and household contacts were reclassified using date of symptom onset, to identify any primary cases who were initially recruited as contacts, and when secondary cases were due to household transmission. We included households with two or more household members. The probable or confirmed case within the household with the earliest onset date was defined to be the primary household case. When two or more household members had the same earliest symptom onset dates these were defined as co-primary cases, as was any case with symptom onset the day after a primary case. All other subjects with later symptom onset dates were defined as secondary cases, apart from those who had symptom onset dates more than 14 days after the primary case.

We performed initial descriptive analyses to explore the characteristics of the contacts. Symptomatic SAR and odds ratios (OR) for secondary transmission were estimated for a range of factors using univariate analyses and multivariate mixed effects logistic regression models with a random intercept for households. The following potential explanatory variables were examined: household size, characteristics of the contact including sex and age group, and characteristics of the index case including sex, age, whether the case was admitted to hospital and whether the symptoms included coughing or sneezing. Adjusted marginal SAR were estimated for each explanatory variable with robust standard errors. Presence or absence of comorbidities among the primary case and contacts were explored as interaction terms.

For the primary analyses, co-primaries were excluded and SAR were based on confirmed and probable secondary cases. Three sensitivity analyses were undertaken: (i) with co-primaries included, (ii) restricted to laboratory-confirmed secondary cases only and (iii) with possible, probable and confirmed secondary cases, the former including those who developed any non-respiratory symptoms (e.g. nausea, fatigue and joint aches) within 14 days of exposure.

Serial interval was defined as time from onset of first symptom in the primary case to time of onset of first symptom in the secondary case with a cut-off of 14 days. We considered the same explanatory variables as in the SAR analysis. We added a lag factor to account for cases who were not present in the household at the time the symptoms of the corresponding index cases started. We also adjusted for the number of cases in the household at the time of first exposure. Individual variables were initially explored using Kaplan–Meier estimates of the survival function. Survival regression was then undertaken using the best fitting of the log-normal, Gamma or Weibull distributions.

The individual basic reproduction number (R_0_) was estimated using the exponential growth model described by Wallinga and Lipsitch and the renewal equation model described by Fraser with adjustment for the contribution of imported cases [16,17]. We used the approach described by Fraser to estimate the household reproduction number (defined as the number of households infected by each infected household) [17]. For estimates of reproduction number, analyses were restricted to cases from the very early stages of the pandemic when all identified cases were included in the FF100.

#### Community contacts

The median incubation period was estimated for probable secondary cases and confirmed secondary cases who had a point source exposure. Exposures before the onset date in the index case were excluded. The timing of exposure among these cases was compared with timing of exposure among contacts who did not develop symptoms. Healthcare workers, returning travellers, airplane exposures and those who had contact with multiple cases were excluded from this analysis.

### Ethical statement

This was an observational surveillance system carried out under the permissions granted under Regulation 3 of The Health Service (Control of Patient Information) Regulations 2020 and under Section 251 of the NHS Act 2006.

## Results

### Characteristics of cases

The initial FF100 dataset consisted of 379 confirmed COVID-19 cases, 357 from England, 19 from Scotland and three from Wales, who developed symptoms between 24 January and 13 March 2020. Of these cases, 199 were imported, 92 were secondary and 88 indigenous. There were slightly more male (56.7%) than female cases among the UK FF100 cases. Cases had a mean age of 47.7 years (standard deviation (SD): 17.4) and ranged between 11 months and 94 years. We have previously reported details of these cases and their outcomes [13].

### Recruitment and follow-up of households

After reclassification, there were 365 primary/co-primary cases residing in 329 households. In 96 households, the case was the only recorded resident. The remaining 269 primary/co-primary cases resided in 233 homes. Thirty-two households had two co-primary cases and two households had three co-primary cases. In 10 households, the primary/co-primary case was younger than 19 years. We identified 472 household contacts; of these, 32 (6.8%) were lost to follow-up, however 11 were linked to testing data (Figure). A total of 135 household contacts developed either cough, fever or anosmia. Among those contacts tested after symptom onset with complete information on onset and test date, the mean time from onset of symptoms to testing was 2.9 days.

**Figure:**
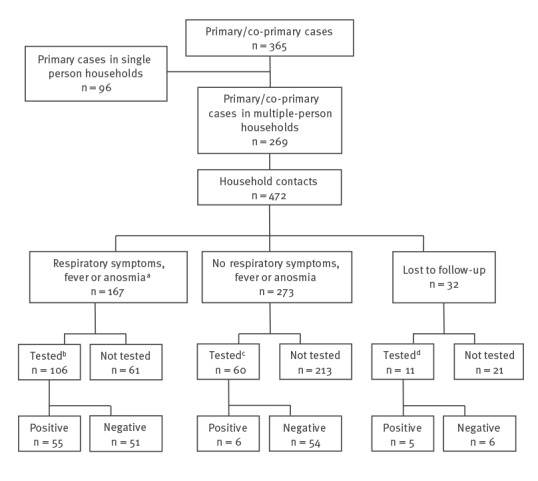
Flowchart of COVID-19 case-patients and household contacts, United Kingdom, 2020 (n = 365 primary cases)

COVID-19: coronavirus disease.

^a ^16 persons had onset of symptoms > 2 weeks after onset date in the primary case.

^b^ Nine persons had specimen date (or laboratory result date if specimen date not known) > 2 weeks after primary case-patient symptoms onset and four had a positive test result.

^c^ Two persons (neither positive) had specimen date (or laboratory result date if specimen date not known) > 2 weeks after primary case-patient symptoms onset.

^d^ One person (not positive) had laboratory result date > 2 weeks after primary case-patient symptoms onset.

The chart includes contacts with any respiratory symptoms, and contacts with at least one of cough, fever or anosmia, contacts from whom specimens were collected and RT-PCR result.

### Household contact characteristics

Characteristics of the household contacts are shown in Table 1. Household size ranged from two to seven people. The age of household contacts ranged from 3 months to 84 years, with a mean age of 29.7 years (SD: 19.9 years), and 241 (51.1%) were female. Comorbidity data was wholly or partially present for 437 household contacts, 60 (13.7%) of whom had an underlying health condition and seven (1.6%) of whom had multimorbidity. The most frequent conditions were asthma and other respiratory disease.

**Table 1:** Characteristics of COVID-19 households and household contacts, United Kingdom, January to March 2020 (n = 472 contacts)

	Non-cases^a^		Probable secondary cases		Confirmed SARS-CoV-2		All	
	n	%	n	%	n	%	n	%
Total	311		96		65		472	
Lost to follow-up								
Yes	24	7.7	3	3.1	5	7.7	32	6.8
No	287	92.3	93	96.9	60	92.3	440	93.2
Household size (number of people)								
2	39	12.5	17	17.7	21	32.3	77	16.3
3	63	20.3	25	26.0	18	27.7	106	22.5
4	104	33.4	30	31.2	18	27.7	152	32.2
5	61	19.6	18	18.8	7	10.8	86	18.2
6	22	7.1	4	4.2	1	1.5	27	5.7
7	22	7.1	2	2.1	0	0.0	24	5.1
Age (years)								
<18	126	40.5	33	34.4	9	13.8	168	35.6
18–64	173	55.6	63	65.6	50	76.9	286	60.6
≥ 65	12	3.9	0	0.0	6	9.2	18	3.8
Sex								
Female	161	51.8	46	47.9	34	52.3	241	51.1
Male	150	48.2	50	52.1	31	47.7	231	48.9
Comorbidities								
Any comorbidity^b^	30	9.6	13	13.5	17	26.2	60	12.7
Asthma requiring medication	13	4.2	2	2.1	10	15.4	25	5.3
Respiratory disease excluding asthma	5	1.6	3	3.1	2	3.1	10	2.1
Diabetes	4	1.3	2	2.1	1	1.5	7	1.5
Heart disease	5	1.6	1	1.0	1	1.5	7	1.5
Immunodeficiency	4	1.3	1	1.0	1	1.5	6	1.3
Malignancy	0	0.0	3	3.1	2	3.1	5	1.1
Neurological disease	2	0.6	1	1.0	2	3.1	5	1.1
Kidney disease	2	0.6	1	1.0	1	1.5	4	0.8
No comorbidity	252	81.0	80	83.3	45	69.2	377	79.9
Unknown	29	9.3	3	3.1	3	4.6	35	7.4

COVID-19: coronavirus disease.

^a^ Non-cases include household contacts with no respiratory symptoms, contacts who developed respiratory symptoms > 14 days after symptom onset in the primary case, and contacts with unrelated prior illnesses (identified through review of household symptomology dates and HPZone case notes).

^b^ Seven household contacts had multimorbidity, therefore the total of the number of contacts with each individual comorbidity will sum to more than the total number of contacts with ‘Any comorbidity’.

Among the 440 contacts with complete follow-up, the most common symptom was cough (n = 116; 26.4%), followed by fatigue (n = 93; 21.1%) and headache (n = 85; 19.3%) (see Supplement 3, Figure S1 for a breakdown of the proportion of household contacts reporting each symptom). A response for anosmia was present for 287 contacts, of these 30 (10.5%) experienced anosmia. Of the contacts who developed fever, cough or anosmia, 68.1% (92/135) were PCR-tested, with 54.3% (50/92) testing positive. Within the follow-up period, 3.6% (16/440) of contacts were hospitalised, with a median duration of stay of 3.5 days (interquartile range: 2–9.5 days). All hospitalised contacts tested positive for SARS-CoV-2. None of the contacts with complete follow-up died during the study period.

### Household transmission dynamics

#### Secondary attack rates

The household symptomatic SAR was 37% (95% confidence interval (CI): 31–43) including both confirmed and probable secondary cases. If restricted to confirmed secondary cases only, the SAR was 16% (95% CI: 11–20) and when possible, probable and confirmed secondary cases were included, the SAR was 43% (95% CI: 37–49).

Unadjusted SAR and OR of probable and confirmed secondary cases by a range of explanatory variables are shown in Table 2 and the multivariate analysis is shown in Table 3. In both the univariate and multivariate analyses, there was an inverse relationship between household size and SAR, with the highest SAR in households with two people (SAR = 0.48; 95% CI: 0.36–0.60) and the lowest in households of five or more (SAR = 0.22; 95% CI: 0.11–0.33). There were no significant effects of sex or presence of comorbidities in either primary cases or contacts nor of presence of cough or sneezing as a symptom in the primary case. The SAR were lowest in contacts younger than 18 years or 65 years and older; however, these effects were not significant. The SAR were highest where the primary case was younger than 18 years with significantly higher odds of secondary infection (OR = 41.89; 95% CI: 4.46–393.02); however, there were only three households with no co-primaries and a primary case aged under 18 years and there is a lot of uncertainty in this finding. Where the primary case was admitted to hospital, the odds of secondary infection in the household were significantly lower (OR = 0.37; 95% CI: 0.18–0.77).

**Table 2:** Unadjusted symptomatic secondary attack rates and odds ratios for secondary SARS-CoV-2 infection (probable and confirmed secondary cases), United Kingdom, January to March 2020 (n =440)

	Variable levels	SAR	95% CI	OR	95% CI
Household					
Household size	2	0.49	0.37–0.60	1.00	Reference value
	3	0.41	0.30–0.51	0.62	0.26–1.53
	4	0.32	0.22–0.42	0.36	0.15–0.90
	≥ 5	0.25	0.13–0.37	0.23	0.07–0.71
Interactions					
Sex	Male → Male	0.37	0.26–0.48	1.00	Reference value
	Male → Female	0.42	0.33–0.50	1.36	0.69–2.69
	Female → Male	0.34	0.26–0.43	0.85	0.34–2.13
	Female → Female	0.29	0.16–0.42	0.59	0.19–1.84
Comorbidities	None → none	0.37	0.29–0.45	1.00	
	None → comorbidities	0.46	0.30–0.62	1.70	0.58–4.95
	Comorbidities → none	0.36	0.25–0.47	0.95	0.39–2.29
	Comorbidities → comorbidities	0.46	0.28–0.64	1.71	0.53–5.56
Characteristics of contact					
Sex	Male	0.36	0.29–0.43	1.00	Reference value
	Female	0.38	0.31–0.45	1.15	0.68–1.94
Age group (years)	< 18	0.30	0.21–0.39	0.62	0.28–1.38
	18–34	0.37	0.28–0.47	1.00	Reference value
	35–64	0.43	0.35–0.51	1.38	0.67–2.85
	≥ 65	0.35	0.13–0.57	0.86	0.19–3.87
Characteristics of primary case					
Sex	Male	0.41	0.33–0.49	1.00	Reference value
	Female	0.33	0.25–0.41	0.61	0.30–1.26
Age group (years)	< 18	0.89	0.7–1.05	41.89	4.46–393.02
	18–64	0.35	0.29–0.41	1.00	
	≥ 65	0.42	0.25–0.59	1.51	0.53–4.26
Hospital admission	Without hospital admission	0.43	0.36–0.51	1.00	Reference value
	With hospital admission	0.28	0.20–0.36	0.37	0.18–0.77
Cough sneezing	Without cough/sneeze	0.32	0.19–0.46	1.00	
	With cough/sneeze	0.38	0.32–0.44	1.42	0.54–3.70
Overall		0.37	0.31–0.43	NA	

CI: confidence interval; NA: not applicable; OR: odds ratio; SAR: secondary attack rate; SARS-CoV-2: severe acute respiratory syndrome coronavirus 2.

**Table 3:** Adjusted symptomatic secondary attack rates and odds ratios for secondary SARS-CoV-2 infection (probable and confirmed secondary cases), United Kingdom, January to March 2020 (n =440)

Variable	Levels	SAR	95% CI	Adjusted OR	95% CI
Household					
Household size	2	0.48	0.36–0.60	1.00	Reference value
	3	0.40	0.29–0.52	0.67	0.28–1.60
	4	0.33	0.23–0.44	0.46	0.19–1.10
	≥ 5	0.22	0.11–0.33	0.22	0.076–0.66
Characteristics of contact					
Sex	Male	0.36	0.28–0.43	1.00	Reference value
	Female	0.32	0.24–0.39	0.80	0.46–1.40
Age group (years)	< 18	0.29	0.19–0.39	0.73	0.32–1.60
	18–34	0.34	0.24–0.44	1.00	Reference value
	35–64	0.39	0.31–0.47	1.30	0.65–2.70
	≥ 65	0.26	0.03–0.50	0.62	0.12–3.30
Characteristics of primary case					
Sex	Male	0.38	0.29–0.46	1.00	Reference value
	Female	0.29	0.21–0.37	0.60	0.30–1.20
Age group (years)	< 18	0.92	0.80–1.00	61	7–527
	18–64	0.31	0.25–0.37	1.00	Reference value
	≥ 65	0.38	0.17–0.58	1.40	0.44–4.70
Hospital admission	Without hospital admission	0.40	0.32–0.48	1.00	Reference value
	With hospital admission	0.25	0.17–0.33	0.40	0.20–0.80
Cough/sneeze	No cough/sneeze	0.29	0.18–0.41	1.00	Reference value
	Cough/sneeze	0.34	0.28–0.41	1.40	0.61–3.00

CI: confidence interval; OR: odds ratio; SAR: secondary attack rate; SARS-CoV-2: severe acute respiratory syndrome coronavirus 2.

When co-primary cases were included in the analysis, results were broadly similar: this increased the number of households with those under 18 years as a primary and the odds of secondary infection remained significant (AOR = 8.00; 95% CI: 0.81-79.00) (see Supplement 3, Table S1 for the results of the secondary analysis including co-primary cases). In the analysis restricted to laboratory-confirmed secondary cases, there is a significantly lower odds of secondary infection in contacts younger than 18 years (OR = 0.22; 95% CI: 0.01–0.88) and the higher odds of secondary infection where the primary case is aged < 18 years remains (OR = 22.00; 95% CI: 4.50-106.00) (see Supplement 3, Table S2 for the results of the secondary analysis restricted to confirmed cases).

#### Serial interval in households

The Weibull distribution provided the best fit for the univariate survival analysis and gave a mean serial interval of 4.67 days (the full distribution and other models tested are provided in Supplement 3, Figure S2 and the model parameters in Supplement 3, Table S3). In the multivariate analysis, explanatory variables that were associated with a shorter serial interval included the primary case experiencing cough as a symptom and the primary case being an imported case (Table 4). There were non-linear relationships between the age of primary case or household contact and the serial interval. The serial intervals were shorter if the primary case was younger than 18 years or an older adult compared with working age adults, and serial interval were longer among household contacts who were those aged under 18 years or older adults compared with working age adults (Supplement 3, Figure S3 shows the effect of age of primary case and contact on serial interval). Crude serial intervals and modelled effect of age as a continuous variable are provided in Supplement 3, Table S4 and Supplement 3, Figure S2, respectively. Supplement 3, Table S5 shows a breakdown of serial intervals by age group of the primary and secondary case, where generally longer serial intervals were seen as age group of the primary case increased.

**Table 4:** Adjusted serial intervals using marginal means and hazard ratios, SARS-CoV-2 infection, United Kingdom, January to March 2020 (n =440)

Variable	Levels	Serial interval	95% CI	HR	95% CI
Primary case					
Imported	No	5.99	4.67–7.69	1.00	
	Yes	4.75	3.57–6.33	1.40	0.96–2.04
Cough	No	6.79	4.96–9.30	1.00	
	Yes	5.04	3.96–6.41	1.55	1.06–2.26
Fever	No	4.50	3.48–5.81	1.00	
	Yes	5.73	4.42–7.43	0.70	0.50–1.00
Age group (years)	< 18	4.04	2.73–5.96	1.50	0.89–2.54
	18–64	5.34	4.22–6.75	1.00	
	≥ 65	6.59	3.82–11.38	0.73	0.35–1.54
Sex	Male	5.00	3.93–6.37	1.00	
	Female	5.82	4.30–7.89	0.80	0.54–1.18
Contact					
Age group (years)	< 18	5.14	4.33–6.10	1.06	0.70–1.59
	18–34	5.34	4.22–6.75	1.00	
	35–64	4.48	3.60–5.58	1.29	0.87–1.91
	≥ 65	5.63	2.53–12.5	0.93	0.29–2.96

CI: confidence interval; HR: hazard ratio; SAR: secondary attack rate; SARS-CoV-2: severe acute respiratory syndrome coronavirus 2.

#### Basic and household reproduction number

Using the approach by Wallinga and Lipsitch, and based on the serial interval above, we obtained an estimate of R_0_ = 3.67 (95% CI: 3.22–3.98) [16]. Using the renewal equation to take into account the contribution of imported cases, the individual R_0_ in the early stages of the pandemic in the UK was estimated at 1.85 (95% CI: 1.20–3.42). Applying the household transmission model to the household data, the average total number of cases in an infected household was 1.67. We estimated the household reproduction number from the same models at 2.33 (95% CI: 1.30–4.89).

### Community contacts

We identified 45 confirmed or probable secondary cases who had a point source exposure (exposure window of maximum 1 day) to a primary case in the FF100 dataset; of these, 12 were laboratory-confirmed secondary cases. The mean incubation period for confirmed and probable cases with a point source exposure was 4.51 days (SD: 2.66), for confirmed secondary cases alone it was 4.77 days (SD: 2.34) (Table 5). Probable and confirmed secondary cases were exposed a mean of 2.37 days (SD: 3.36) after symptom onset in the primary case, ranging from 0 to 14 days. Restricting the analysis to confirmed secondary cases alone, exposure occurred a mean of 1.33 days (SD: 1.61) after symptom onset in the primary case, ranging from 0 to 5 days. This compares to 2.71 days among contacts who did not become cases. Further details of the timing of onset and exposure for the confirmed secondary cases are shown in Supplement 3, Figure S4.

**Table 5:** Summary of incubation period and timing of exposure in relation to primary COVID-19 case symptom onset for contacts with a point source exposure, United Kingdom, January to March 2020 (n = 286)

		Incubation period (days)					Timing of exposure after symptom onset of primary case (days)				
Status of contact	Number of contacts included	Mean	SD	Median	IQR	Range	Mean	SD	Median	IQR	Range
Probable and confirmed secondary cases	45^a^	4.51	2.66	4.00	2.00–7.00	0.00–11.00	2.37	3.36	1.00	0.00–4.00	0.00–14.00
Confirmed secondary cases	12	4.75	2.34	4.00	3.75–5.00	2.00–11.00	1.33	1.61	1.00	0.00–1.25	0.00–5.00
Did not develop symptoms	241	NA			NA	NA	2.71	2.74	2.00	0.00–5.00	0.00–9.00

COVID-19: coronavirus disease; IQR: interquartile range; NA: not applicable; SD: standard deviation.

^a^ Four persons were excluded from timing of exposure analysis because no onset date was available for the primary case.

## Discussion

In the UK, before the implementation of physical distancing measures, we estimated an overall household symptomatic SAR of 37%, a serial interval of 4.67 days, an R_0_ of 1.85 and a household reproduction number of 2.33. Symptomatic secondary attack rates were lower in larger households. There is some suggestion that where the primary case was younger than 18 years, household SAR were higher and the serial interval was shorter. Conversely, serial intervals were longer if the household contact was younger than 18 years or an older adult. Using point source exposures, we estimated a mean incubation period of around 4.5 days.

Our estimated household SAR in the UK was greater than that reported in China, Taiwan and South Korea where the estimated household SAR during the first 3 months of the pandemic ranged from 5% to 30% [4-10]. Making comparisons across studies is challenging because of differences in follow-up, symptom ascertainment or testing of contacts, however, the higher household SAR in the UK could reflect differences in isolation and infection control measures taken to reduce spread. In the UK, individuals meeting the case definition were advised to minimise contact with others in the household, wash hands regularly and cover coughs and sneezes. This is broadly similar to advice issued elsewhere, although more stringent advice on quarantine within the household and wearing masks was in place in some areas, and cases were taken out of the household and placed in isolation facilities [4,8]. It is also possible that timing of the diagnosis of secondary cases was more delayed in the very early stages of the pandemic in China and other countries that experienced early cases, when less was understood about the disease. Our serial interval estimate is broadly similar to previous estimates from Asia that range from 4.0 to 6.3 days [4,18-20].

This high estimated R_0_ using the Wallinga and Libsitch approach is likely to be a result of the method neglecting the contribution of continuously imported cases to transmission dynamics in UK. [16] After adjusting for this contribution, the R_0_ was lower than existing estimates obtained for the early stage in China, although confidence intervals overlapped [21-24].

We estimated a mean incubation period of 4.5–4.8 days, slightly lower than previous estimates which ranged from 5.5 to 6.4 days [25-27]. Previous estimates have been based on estimated distributions using earliest and latest exposure period. We restricted our analysis to those with unique point source exposures to allow us to precisely estimate exposure date. The incubation period ranged from 2 to 11 days for confirmed secondary cases and 0–11 days for probable and confirmed cases combined, suggesting that advice from March 2020 of isolation of contacts for 14 days after exposure is sufficient and could potentially be lowered to 11 days. Consideration may also be given to defining the period of isolation based on timing of symptom onset in the first case. The mean time from onset in the primary case to exposure among confirmed secondary cases was 1.33 days, suggesting that cases are most infectious soon after symptom onset. However, it should be noted that, at the time, contact tracing was only undertaken from the time of symptom onset in the primary case, not before symptom onset, therefore transmission prior to symptom onset may not have been captured. This could reduce the mean time from symptom onset to exposure.

A systematic review and meta-analysis by Viner et al. examined susceptibility of children to SARS-CoV-2 and their role in transmission [28]. The pooled estimated odds of children being an infected contact, compared with adults, were 0.44 (95% CI: 0.29–0.69). When restricted to household transmission studies alone, the pooled estimate was 0.19 (95% CI: 0.10–0.36). While we saw lower SAR among contacts younger than 18 years, this was only significant in the analysis that was restricted to confirmed secondary cases. When probable secondary cases were included, the effect was no longer significant. This may reflect milder symptoms and a lower propensity for testing those aged under 18 years, in which case previous estimates, with more stringent case definitions, in particular those relying on PCR-confirmed cases alone, would underestimate SAR in those aged under 18 years. The review found no studies that reported SAR where children or adolescents were the primary cases. A review of household clusters by Zhu et al. found that only three of 31 household transmission clusters had a child as the index case, and suggested that children do not play a substantial role in transmission [29]. Nevertheless, the low number of households with children as the index may be due to lower ascertainment in children if they are less likely to present with symptoms [30]. However, recent evidence suggests that children carry higher levels of SARS-CoV-2 genetic material in their nose and throat than adults, which would support our findings of a higher symptomatic secondary attack rate among household contacts of children [31]. Furthermore, a recent study from South Korea found that the highest proportion of positive household contacts was among contacts of index cases aged 10–19 years [32]. Nevertheless, the South Korean study did not identify whether index cases were the primary cases, therefore we do not know the direction of transmission.

Our study has a number of strengths: this was one of the largest COVID-19 household studies in the early phase of the pandemic and one of the only studies outside of Asia. Data were collected through direct patient interviews and high rates of follow-up were achieved with good data completeness, and household contacts were actively followed up by local health protection teams on a daily basis to monitor symptoms. We identified point source case–secondary case pairs, which allowed us to directly estimate the incubation period without having to model timing of infection. 

The study also has a number of limitations: test results were not available for some participants who developed symptoms, therefore we probably under-ascertained confirmed secondary cases. Furthermore, as with previous studies, testing was focussed on those who developed symptoms. Estimates of asymptomatic infection range from 4% to 41%, therefore we are likely to have missed asymptomatic cases [33]. Furthermore, rates of asymptomatic infection appear to be highest in children, therefore we particularly underestimated secondary infection rates in those younger than 18 years [30,33]. We are currently have also undertaken further analyses of household transmission incorporating swabbing of asymptomatic contacts and serology which will provide a better understanding of true secondary infection rates and asymptomatic infection [34]. We are also limited in our ability to draw any clear conclusions about transmission from those aged under 18 years because the number of households with primary cases younger than 18 years was small. Similarly, the breakdown of serial interval by age of the primary case and secondary case lacks precision because of small numbers in some age groups.

## Conclusion

Since the early stages of the pandemic, data from the FF100 study have been shared in real time with independent modelling groups advising governments, and they have informed policymaking and public health management guidance. The high household SAR and the lack of transmission in a range of other settings highlight the importance of the household setting for onwards transmission. This emphasises the need for hygiene measures within the household and, where vulnerable people are involved, maintaining distancing within the household, in particular if a household member develops symptoms. While the numbers were small, the high household SAR from paediatric primary cases suggest that high rates of transmission are seen in all ages and further consideration is needed as to whether this may apply to settings outside the household.

## Supplementary Data

538000 Supplement1

## Supplementary Data

240000 Supplement2

## Supplementary Data

343000 Supplement3
