# Supplementary material for: Transmission dynamics of COVID-19 in household and community settings in the United Kingdom, January to March 2020
Source: Euro Surveill. 2022 Apr 14;27(15):2001551. doi: 10.2807/1560-7917.ES.2022.27.15.2001551 (PMC9012093; doi:10.2807/1560-7917.ES.2022.27.15.2001551)
Supplement: Supplement1 [file 20-01551_BERNAL_Supplement1_Questionnaire1.pdf]

Supplement 1

This supplementary material is hosted by *Eurosurveillance* as supporting information alongside the article [Transmission dynamics of COVID-19 in household and community settings in the United Kingdom, January to March 2020] on behalf of the authors who remain responsible for the accuracy and appropriateness of the content. The same standards for ethics, copyright, attributions and permissions as for the article apply. Supplements are not edited by *Eurosurveillance* and the journal is not responsible for the maintenance of any links or email addresses provided therein.

For reference only					
Confirmed case number (issued by National i.e NIS HPZone)		Local ID of contact (local HPZone ID for contact)		FF100 id number (automatically generated on FF100 web tool)	
Name of confirmed case (lab confirmed case that this person is a contact of)					

*Mandatory fields in pink with bold text and an asterisk	
**1. Reporter Details**	
***Name of the reporter (person completing the form)**	
***Date Reported**	
Position	
Organisation	
Phone and extension	
Mobile	
Email	
Fax	
***Date of interview with informant**	

2. Informant Details				
Informant (Please mark x)	Contact		Other	
If Other:				
Contact details including telephone number				
Relationship with contact				

3. Details of the Contact				
NHS number				
***Forename**				
***Surname**				
Sex	Male	Female	Not Specified	
Date of Birth			Age	
Local ID Number (HPZone number)				
Street Address			Home Telephone	
Town			Work Telephone	
County			Mobile	
Post Code			Email address	
Preferred mode of contact				
Country of Residence			Nationality	
Country of Birth				
***Responsible PHE Centre**				
Local Authority				
Name of school if appropriate				
***Location of contact with confirmed case**	Household	Health care setting	School	Aircraft
	Other, please specify			
***Date of last contact with confirmed case (i.e. date of exposure (protected or unprotected) if still in contact please put today's date). This date MUST be included for the daily follow up**				
Is the case part of an institutional outbreak?	Yes	No	Unknown	
	If yes, please specify:			

Communications to PHE should only be sent between PHE email addresses.

Occupation				
*Healthcare Worker (HCW) working with confirmed case	Yes	No		
	If Yes HCW, please complete Sections 4,5 and 6			
	If Not HCW, please complete Sections 4,5 and 7			

4. Symptoms in this contact

*Has the contact been ill within the 14 days after exposure tothe confirmed case?	Yes	No		
*Is the contact currently ill?	Yes	No		
	If contact has not been ill please go to Section 5.			
Date of first symptom onset			Unknown (x)	
Maximum temperature				

Respiratory symptoms

Fever or history of Fever (≥38°C)	Yes	If yes, first onset date	No	Unknown
Runny nose	Yes	If yes, first onset date	No	Unknown
Sneezing	Yes	If yes, first onset date	No	Unknown
Cough	Yes	If yes, first onset date	No	Unknown
	If yes, dry or productive?		Dry	Productive
Shortness of breath	Yes	If yes, first onset date	No	Unknown
Sore throat	Yes	If yes, first onset date	No	Unknown

Other symptoms

Diarrhoea	Yes	No	Unknown
Nausea	Yes	No	Unknown
Vomiting	Yes	No	Unknown
Fatigue	Yes	No	Unknown
Muscle ache	Yes	No	Unknown
Joint ache	Yes	No	Unknown
Loss of appetite	Yes	No	Unknown
Headache	Yes	No	Unknown
Seizures	Yes	No	Unknown
Altered Consciousness	Yes	No	Unknown
Nose bleed	Yes	No	Unknown
Rash	Yes	No	Unknown
Other	Yes	No	Please specify

Communications to PHE should only be sent between PHE email addresses.

5. Medical History

Does the contact have any underlying medical conditions? Complete where appropriate

This information is collected to understand whether specific groups are at greater risk of infection with this virus, to inform future health advice and precautions.

Condition	Yes	No	Unknown	Details
Chronic heart disease				
Diabetes				
HIV/other immunodeficiency				
Chronic kidney disease				
Chronic liver disease				
Chronic respiratory disease, excluding asthma requiring medication				
Asthma requiring medication				
Malignancy				
Organ or bone marrow recipient				
Seizure disorder				
Chronic neurological disease				

Currently pregnant	Yes	No	Unknown	
	If yes, trimester	First	Second	Third
	If yes, estimated delivery date			
Height in cm				
Weight in kg				
Other, please specify				

Communications to PHE should only be sent between PHE email addresses.

6. Exposure in a healthcare setting

Was any contact with the confirmed case unprotected (without full PPE)?	Yes	No			
	First date of unprotected contact				
	Last date of unprotected contact				
Was any contact with the confirmed case protected (full PPE)?	Yes	No			
	First date of protected contact				
	Last date of protected contact				
Job title					
Place of work					
Direct patient contact? (eg Hands-on clinical contact)	Yes	No			

What type of protective equipment was used during contac with confirmed case and how often?

Surgical mask	Yes	No	Don't know		
	If yes, how often	Always (100% of time)	Often (>50% of time)	Infrequent (<50% of time)	Never
High filtration mask (FFP3)	Yes	No	Don't know		
	If yes, how often	Always (100% of time)	Often (>50% of time)	Infrequent (<50% of time)	Never
Eye protection	Yes	No	Don't know		
	If yes, how often	Always (100% of time)	Often (>50% of time)	Infrequent (<50% of time)	Never
Gloves	Yes	No	Don't know		
	If yes, how often	Always (100% of time)	Often (>50% of time)	Infrequent (<50% of time)	Never
Gown	Yes	No	Don't know		
	If yes, how often	Always (100% of time)	Often (>50% of time)	Infrequent (<50% of time)	Never
Was the contact present while any aerosol generating procedures took place?	Yes	No			
If yes, what procedure were they present at? List and date if more than one	Procedure 1			Date	
	Procedure 2			Date	
	Procedure 3			Date	
Was the contact wearing any type of mask at this/these procedure?		Surgical	FFP3	None	
	Procedure 1				
	Procedure 2				
	Procedure 3				
Date of illness onset for confirmed case (if known)					

Communications to PHE should only be sent between PHE email addresses.

If date of onset in confimed case is known:
Please mark with x below all days of contact with the confirmed case since onset of illness in the confirmed case.
E.g. 0 means contact on the day of onset of illness. +1 means contact the day after onset of illness
If date of onset in confirmed case is not known please skip to next question

Day	0	1	2	3	4
Date (autocompletes from question above)					
Enter x for contact occurred on this day					
Day	5	6	7	8	9
Date					
Enter x for contact occurred on this day					
Day	10	11	12	13	14
Date					
Enter x for contact occurred on this day					
If date of onset in confirmed case is not known, please give the total number of days you were in contact with the confirmed case while they were symtomatic					
Monitoring state of contact following initial assessment	Active		Passive		

Communications to PHE should only be sent between PHE email addresses.

7. Exposure Information - Non Healthcare Workers					
Has the contact been in continuous contact with the confirmed case? E.g. same household	Yes	No	Unknown		
	If no, date of first contact with case				
Last date of contact with confirmed case			Contact has not ended (x)		
Date of illness onset for confirmed case (if known)					
If date of onset in confimed case is known:<br>Please mark with x below all days of contact with the confirmed case since onset of illness in the confirmed case.<br>E.g. 0 means contact on the day of onset of illness. +1 means contact the day after onset of illness<br>If date of onset in confirmed case is not known please skip to next question					
Day	0	1	2	3	4
Date (autocompletes from question above)					
Enter x for contact occurred on this day					
Day	5	6	7	8	9
Date					
Enter x for contact occurred on this day					
Day	10	11	12	13	14
Date					
Enter x for contact occurred on this day					
If date of onset in confirmed case is not known, please give the total number of days you were in contact with the confirmed case while they were symtomatic					
Monitoring state of contact following initial assessment	Active		Passive		

8. Outcome/Current Status of Contact					
Please complete only if has been ill since exposure or is currently ill.					
Status	Recovered	If recovered. Date symptoms resolved (able to resume normal activities)	ill	Dead	If dead, date of death
Was the contact hospitalised?	Yes	No	Don't know		
If hospitalisation occurred:	Is the contact still hospitalised?	Yes	No	Don't know	
	If yes, date of admission				
	Date of discharge (leave blank if patient is still hospitalised)				
Case classification of contact if appropriate (see below for definitions)	Confirmed	Presumptive positive	Possible	Discarded	N/A
	PHE National Reference Laboratory (RVU) Colindale positive confirmation of infection with novel-CoV	PHE novel-CoV Testing Laboratory positive of infetion with novel-CoV	Acute respiratory infection or more severe illness as per possilbe case algorithm	Any possible case with a negative novel CoV laboratory result	Not applicable

Communications to PHE should only be sent between PHE email addresses.

9. Virological Tests (if appropriate)

Specimen Date	Laboratory Test Date	Specimen Type[3]	Lab Name[4]	Local Lab Number	Virus	Type of Test[5]	Result

[3] Specimen Types: BAL, Blood-Plasma, Blood-Serum, Faeces, Nose/Throat swab, NPA, Sputum, Tissue, Oral fluid, Finger prick, Urine, Endotracheal aspirate

[4] Lab Names: Belfast, Birmingham, Bristol, Cambridge, Cardiff, Dublin, Glasgow, Leeds, Leicester, Liverpool, London-Barts, London-Kings, London-St Thomas's, London-UCLH, Manchester, Newcastle, Nottingham, PHE-RVU, Porton, Southampton, Other (please specify)

[5] Test Types: RT-PCR, Sequencing, Pyrosequencing, Antigenic-typing, Phenotypic antiviral susceptibility testing, HA/MN

10. Serology

	Yes	No	Not sure
Has baseline serology been taken on contact?			
If yes, date serology taken?			
Laboratory Name			
Date serology sent to a PHE lab			

Communications to PHE should only be sent between PHE email addresses, directed to respiratory.lead@phe.gov.uk

PHE strives to comply with all data protection legislation and confidentiality best practice in accordance with the Data Protection Act (2018) and the General Data Protection Regulation (GDPR). All data will be handled securely. At local level data shared electronically will be from secure nhs.net to nhs.net email accounts. Communications to PHE (between HPTs and authorised PHE National Infection service staff) will be between secure phe.gov.uk accounts. Data is held by PHE NIS is in a secure web system hosted on encrypted NHS N3 network and access to this database is restricted to authorised scientific and medical staff.

Data collection is undertaken with permissions granted under Regulation 3 of The Health Service (Control of Patient Information) Regulations 2002, and without explicit patient permission under Section 251 of the NHS Act 2006 (http://www.legislation.gov.uk/uksi/2002/1438/regulation/3/made).
