# Supplementary material for: Transmission dynamics of COVID-19 in household and community settings in the United Kingdom, January to March 2020
Source: Euro Surveill. 2022 Apr 14;27(15):2001551. doi: 10.2807/1560-7917.ES.2022.27.15.2001551 (PMC9012093; doi:10.2807/1560-7917.ES.2022.27.15.2001551)
Supplement: Supplement2 [file 20-01551_BERNAL_Supplement2_Questionnaire2.pdf]

Supplement 2

This supplementary material is hosted by *Eurosurveillance* as supporting information alongside the article [Transmission dynamics of COVID-19 in household and community settings in the United Kingdom, January to March 2020] on behalf of the authors who remain responsible for the accuracy and appropriateness of the content. The same standards for ethics, copyright, attributions and permissions as for the article apply. Supplements are not edited by *Eurosurveillance* and the journal is not responsible for the maintenance of any links or email addresses provided therein.

Contact Follow-up Form - 2b
v6- COVID-19

For reference only					
Confirmed case number (issued by National i.e NIS HPZone)		Local ID of contact (local HPZone ID for contact)		FF100 id number (automatically generated on FF100 web tool)	
Name of confirmed case					

***Mandatory fields in pink with bold text and an asterisk**
Questions in italics only need to be updated if information has changed since the 2a form

1. Details of the Contact	
NHS number	
***Forename**	
***Surname**	
***Date of birth**	

2. Reporter Details (if different to 2a)	
***Name of the reporter (person completing the form)**	
***Date Reported**	
Position	
Organisation	
Phone and extension	
Mobile	
Email	
Fax	
***Date of interview with informant**	

3. Informant Details (if different to 2a)				
Informant (Please mark x)	Contact		Other	
If Other:				
Contact details including telephone number				
Relationship with contact				

Communications to PHE should only be sent between PHE email addresses.

4. Exposure Information (if different to 2a)					
*Last date of contact with confirmed case (If still in contact put today's date)			Contact has not ended (x)		
Date of illness onset for confirmed case (if known)					
If date of onset in confimed case is known:<br>Please mark with x below all days of contact with the confirmed case since onset of illness in the confirmed case.<br>E.g. 0 means contact on the day of onset of illness. +1 means contact the day after onset of illness<br>If date of onset in confirmed case is not known please skip to next question					
Day	0	1	2	3	4
Date (autocompletes from question above)					
Enter x for contact occurred on this day					
Day	5	6	7	8	9
Date					
Enter x for contact occurred on this day					
Day	10	11	12	13	14
Date					
Enter x for contact occurred on this day					
If date of onset in confirmed case is not known, please give the total number of days you were in contact with the confirmed case while they were symtomatic					

Communications to PHE should only be sent between PHE email addresses.

4. Symptoms in this contact (final updated)					
*Has the contact been ill within the 14 days after exposure to the confirmed case? (see symptoms)	Yes	No			
*Is the contact currently ill?	Yes	No			
Did the contact have any additional symptoms not previously mentioned in form 2a and up to 14 days since last exposure with confirmed case?					
If yes, date of first symptom onset				Unknown (x)	
If yes, time of onset	AM	PM	Unknown		
If yes, maximum temperature (°C)					
Respiratory symptoms					
Fever or history of Fever (≥38°C)	Yes	If yes, first onset date	No	Unknown	
Runny nose	Yes	If yes, first onset date	No	Unknown	
Sneezing	Yes	If yes, first onset date	No	Unknown	
Cough	Yes	If yes, first onset date	No	Unknown	
	If yes, dry or productive?		Dry	Productive	
Shortness of breath	Yes	If yes, first onset date	No	Unknown	
Sore throat	Yes	If yes, first onset date	No	Unknown	

Communications to PHE should only be sent between PHE email addresses.

Other symptoms					
Diarrhoea	Yes	No	Unknown		
Nausea	Yes	No	Unknown		
Vomiting	Yes	No	Unknown		
Fatigue	Yes	No	Unknown		
Muscle ache	Yes	No	Unknown		
Joint ache	Yes	No	Unknown		
Loss of appetite	Yes	No	Unknown		
Headache	Yes	No	Unknown		
Seizures	Yes	No	Unknown		
Altered Consciousness	Yes	No	Unknown		
Nose bleed	Yes	No	Unknown		
Rash	Yes	No	Unknown		
Other	Yes	No	Please specify		
Loss of sense of smell	Yes	If yes, first onset date	No	Unknown	
If yes has sense of smell returned?	Yes	If yes, first onset date	No	Unknown	

Communications to PHE should only be sent between PHE email addresses.

5. Outcome/Current Status of this contact

Please complete only if has been ill since exposure or is currently ill.

Status	Recovered	If recovered. Date symptoms resolved (able to resume normal activities)	ill	Dead	If dead, date of death
Was the case hospitalised?	Yes	No	Don't know		
If hospitalisation occurred:	Is the contact still hospitalised?	Yes	No	Don't know	
	If yes, date of admission				
	Date of discharge (leave blank if patient is still hospitalised)				
If Dead: (n.b. If this information is not currently available, please leave blank and send through an update as soon as results are known)	Contribution of WN-CoV to death (mark x in one only)	Underlying/primary			
		Contributing/secondary			
		No contribution to death			
		Unknown			
	Was apost mortem performed?	Yes	No	Don't know	
	Cause of death as MCCD (Medical Certificate of the cause of death)				
	Result of coroner's report where applicable				

Communications to PHE should only be sent between PHE email addresses.

6. Serology

Has convalescent serology been taken on contact?	Yes	No	Not sure
If yes, date serology taken?			
Laboratory Name			
Date serology sent to a PHE lab			

7. Final contact classification

*MANDATORY: Please mark only one box in this section

If contact becomes a possible case: (see below for definitions)	Confirmed	Presumptive positive	Possible	Discarded
	PHE National Reference Laboratory (RVU) Colindale positive confirmation of infection with novel-CoV	PHE novel-CoV Testing Laboratory positive of infetion with novel-CoV	Acute respiratory infection ormore severe illness as per possilbe case algorithm	Any possible case with a negative novel CoV laboratory result
Asymptomatic contact: No illness<br><br>Contact did not become ill during the 14 days after case exposure (protected/unprotected contact)				
Lost to follow up<br><br>If the contact is lost (non-responsive) during the follow up period				

Communications to PHE should only be sent between PHE email addresses, directed to respiratory.lead@phe.gov.uk

PHE strives to comply with all data protection legislation and confidentiality best practice in accordance with the Data Protection Act (2018) and the General Data Protection Regulation (GDPR). All data will be handled securely. At local level data shared electronically will be from secure nhs.net to nhs.net email accounts. Communications to PHE (between HPTs and authorised PHE National Infection service staff) will be between secure phe.gov.uk accounts. Data is held by PHE NIS is in a secure web system hosted on encrypted NHS N3 network and access to this database is restricted to authorised scientific and medical staff.

Data collection is undertaken with permissions granted under Regulation 3 of The Health Service (Control of Patient Information) Regulations 2002, and without explicit patient permission under Section 251 of the NHS Act 2006 (http://www.legislation.gov.uk/uksi/2002/1438/regulation/3/made).
