# Supplementary material for: Transmission dynamics of COVID-19 in household and community settings in the United Kingdom, January to March 2020
Source: Euro Surveill. 2022 Apr 14;27(15):2001551. doi: 10.2807/1560-7917.ES.2022.27.15.2001551 (PMC9012093; doi:10.2807/1560-7917.ES.2022.27.15.2001551)
Supplement: Supplement3 [file 20-01551_BERNAL_Supplement3.pdf]

## Supplement 3

This supplementary material is hosted by *Eurosurveillance* as supporting information alongside the article [Transmission dynamics of COVID-19 in household and community settings in the United Kingdom, January to March 2020] on behalf of the authors who remain responsible for the accuracy and appropriateness of the content. The same standards for ethics, copyright, attributions and permissions as for the article apply. Supplements are not edited by *Eurosurveillance* and the journal is not responsible for the maintenance of any links or email addresses provided therein.

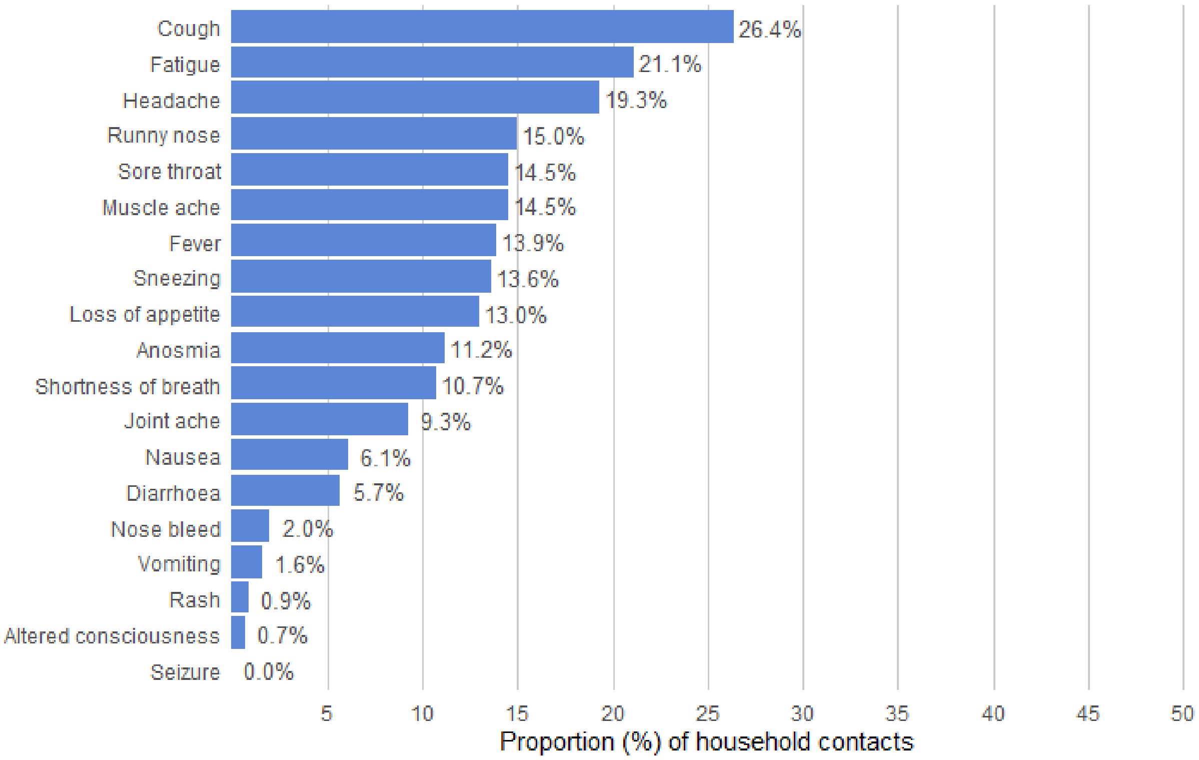


*Supplementary Figure 1: Proportion of household contacts reporting symptoms during follow-up period*

*Supplementary Table 1: Adjusted secondary attack rates and odds ratios for secondary infection (probable and confirmed secondary cases with co-primaries included)*

| | variable | levels | SAR | 95% CI | | OR | 95% CI | |
|---|---|---|---|---|---|---|---|---|
| household | household size | 2 | 0.39 | 0.28 | 0.49 | 1.00 | | |
| | | 3 | 0.36 | 0.26 | 0.46 | 0.84 | 0.38 | 1.90 |
| | | 4 | 0.31 | 0.22 | 0.40 | 0.64 | 0.29 | 1.40 |
| | | >=5 | 0.22 | 0.12 | 0.32 | 0.36 | 0.14 | 0.94 |
| characteristics of contact | gender | Male | 0.32 | 0.25 | 0.39 | 1.00 | | |
| | | Female | 0.29 | 0.22 | 0.36 | 0.83 | 0.49 | 1.40 |
| | age group | <18 | 0.26 | 0.18 | 0.35 | 0.65 | 0.31 | 1.30 |
| | | 18-34 | 0.33 | 0.24 | 0.42 | 1.00 | | |
| | | 35-64 | 0.33 | 0.26 | 0.41 | 1.00 | 0.53 | 1.90 |
| | | 65+ | 0.24 | 0.024 | 0.45 | 0.54 | 0.11 | 2.60 |
| characteristics of primary case | gender | Male | 0.35 | 0.27 | 0.42 | 1.00 | | |
| | | Female | 0.26 | 0.19 | 0.33 | 0.59 | 0.31 | 1.10 |
| | age group | <18 | 0.67 | 0.28 | 1.10 | 8.00 | 0.81 | 79 |
| | | 18-64 | 0.29 | 0.24 | 0.34 | 1.00 | | |
| | | 65+ | 0.38 | 0.18 | 0.58 | 1.70 | 0.55 | 5.30 |
| | hospital admission | without hospital adm. | 0.35 | 0.28 | 0.42 | 1.00 | | |
| | | with hospital adm. | 0.24 | 0.17 | 0.31 | 0.49 | 0.26 | 0.91 |
| | cough/sneeze | no cough/sneeze | 0.25 | 0.14 | 0.35 | 1.00 | | |
| | | cough/sneeze | 0.32 | 0.26 | 0.37 | 1.50 | 0.69 | 3.30 |

*Supplementary Table 2: Adjusted secondary attack rates and odds ratios for secondary infection (confirmed secondary cases only)*

| | variable | levels | SAR | 95% CI | | OR | 95% CI | |
|---|---|---|---|---|---|---|---|---|
| household | household size | 2 | 0.17 | 0.079 | 0.26 | 1.00 | | |
| | | 3 | 0.13 | 0.05 | 0.20 | 0.63 | 0.19 | 2.10 |
| | | 4 | 0.12 | 0.044 | 0.19 | 0.55 | 0.16 | 1.90 |
| | | >=5 | 0.051 | 0 | 0.11 | 0.18 | 0.03 | 1.00 |
| characteristics of contact | gender | Male | 0.11 | 0.056 | 0.16 | 1.00 | | |
| | | Female | 0.094 | 0.041 | 0.15 | 0.79 | 0.30 | 2.10 |
| | age group | <18 | 0.048 | 0.0088 | 0.087 | 0.22 | 0.056 | 0.88 |
| | | 18-34 | 0.14 | 0.054 | 0.22 | 1.00 | | |
| | | 35-64 | 0.14 | 0.076 | 0.20 | 0.97 | 0.34 | 2.80 |
| | | 65+ | 0.30 | 0.031 | 0.57 | 4.20 | 0.48 | 36 |
| characteristics of primary case | gender | Male | 0.12 | 0.055 | 0.18 | 1.00 | | |
| | | Female | 0.085 | 0.038 | 0.13 | 0.62 | 0.21 | 1.80 |
| | age group | <18 | 0.47 | 0.30 | 0.64 | 22.00 | 4.50 | 106 |
| | | 18-64 | 0.096 | 0.052 | 0.14 | 1.00 | | |
| | | 65+ | 0.098 | 0 | 0.20 | 1.00 | 0.19 | 5.40 |
| | hospital admission | without hospital adm. | 0.14 | 0.078 | 0.20 | 1.00 | | |
| | | with hospital adm. | 0.061 | 0.018 | 0.11 | 0.31 | 0.094 | 1.00 |
| | cough/sneeze | no cough/sneeze | 0.035 | 0 | 0.079 | 1.00 | | |
| | | cough/sneeze | 0.12 | 0.07 | 0.17 | 5.60 | 0.95 | 33 |

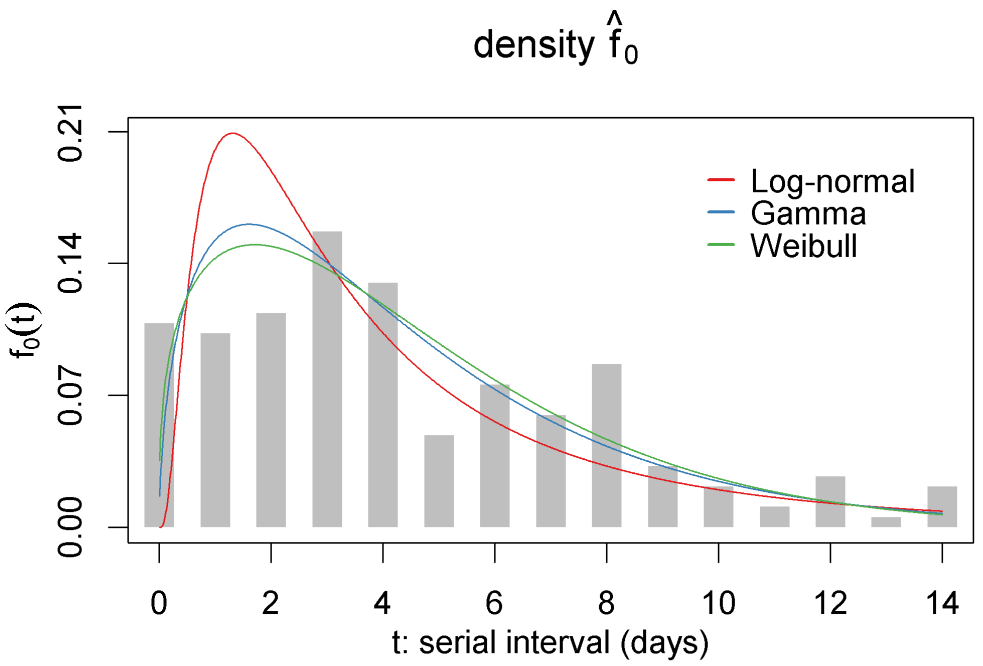


*Supplementary Figure 2: Fit baseline unadjusted density f0(t) on Log-normal, Gamma, Weibull (for households with index case being in the household at the time of onset)*

The parametrisation we follow in this report for Log-normal, Gamma, and Weibull, is given below

$$f_{Log-normal}(t) = \frac{1}{t\sigma\sqrt{2\pi}} exp\left(\frac{-(log(t)\ -\ \mu)^2}{2\sigma^2}\right), \qquad \sigma: scale, \mu: shape$$

$$f_{Gamma}(t) = \frac{\beta^\alpha}{\Gamma(\alpha)} t^{\alpha-1} exp(-\beta t), \qquad \beta: scale, \alpha: shape$$

$$f_{Weibull}(t) = \lambda\gamma(\lambda t)^{\gamma-1} exp(-(\lambda t)^\gamma), \qquad \lambda: scale, \gamma: shape$$

*Supplementary Table 3: parameters for serial interval models*

| | model parameters | | | | | | serial interval | Goodness of fit parametric models | Type of households |
|---|---|---|---|---|---|---|---|---|---|
| | Shape | | | Scale | | | Mean | | |
| Log-normal (unadj) | mu | mu_lb | mu_ub | sigma | sigma_lb | sigma_ub | mean | AIC | All households |
| | 1.15 | 1.01 | 1.30 | 0.94 | 0.86 | 1.02 | 4.90 | 926.19 | |
| Gamma (unadj) | alpha | alpha_lb | alpha_ub | beta | beta_lb | beta_ub | mean | AIC | |
| | 1.56 | 1.29 | 1.87 | 0.35 | 0.28 | 0.43 | 4.50 | 907.42 | |
| Weibull (unadj) | lambda | lambda_lb | lambda_ub | gamma | gamma_lb | gamma_ub | mean | AIC | |
| | 0.20 | 0.18 | 0.23 | 1.33 | 1.19 | 1.48 | 4.50 | 906.01 | |
| Log-normal (unadj) | mu | mu_lb | mu_ub | sigma | sigma_lb | sigma_ub | mean | AIC | Households with index case being in the household at the time of onset |
| | 1.21 | 1.05 | 1.37 | 0.91 | 0.82 | 1.01 | 5.06 | 779.63 | |
| Gamma (unadj) | alpha | alpha_lb | alpha_ub | beta | beta_lb | beta_ub | mean | AIC | |
| | 1.65 | 1.35 | 2.03 | 0.35 | 0.28 | 0.45 | 4.67 | 762.4 | |
| Weibull (unadj) | lambda | lambda_lb | lambda_ub | gamma | gamma_lb | gamma_ub | mean | AIC | |
| | 0.20 | 0.17 | 0.22 | 1.38 | 1.22 | 1.55 | 4.67 | 760.88 | |

*Supplementary Table 4: Crude serial interval, all households*

| | varname | level | serial interval | conf.low | conf.high |
|---|---|---|---|---|---|
| household | household size | 2 | 4.15 | 3.32 | 5.18 |
| | | 3 | 5.88 | 4.72 | 7.33 |
| | | 4 | 4.92 | 4.05 | 5.97 |
| | | 5 | 4.56 | 3.58 | 5.8 |
| primary case | imported | non-imported | 5.52 | 4.67 | 6.52 |
| | | imported | 4.46 | 3.8 | 5.23 |
| | cough | no cough | 5.89 | 4.82 | 7.2 |
| | | cough | 4.68 | 4.09 | 5.36 |
| | fever | no fever | 4.56 | 3.72 | 5.58 |
| | | fever >37C | 5.01 | 4.32 | 5.8 |
| | age group | 18-64 | 4.83 | 4.23 | 5.51 |
| | | <18 | 3.83 | 2.22 | 6.61 |
| | | 65+ | 6.46 | 4.92 | 8.48 |
| | gender | Male | 4.76 | 4.07 | 5.56 |
| | | Female | 5.09 | 4.29 | 6.04 |
| | hospital admission | no hosp. | 4.74 | 4.09 | 5.48 |
| | | hosp. admission | 5.34 | 4.46 | 6.4 |
| | complications | without compl. | 4.72 | 4.14 | 5.37 |
| | | with compl. | 6.33 | 4.87 | 8.23 |
| contact | gender | Male | 4.95 | 4.2 | 5.83 |
| | | Female | 4.85 | 4.14 | 5.68 |
| | age group | 18-34 | 5.11 | 4.13 | 6.31 |
| | | <18 | 5.15 | 4.35 | 6.09 |
| | | 35-64 | 4.51 | 3.78 | 5.39 |
| | | 65+ | 6.84 | 4.69 | 9.97 |

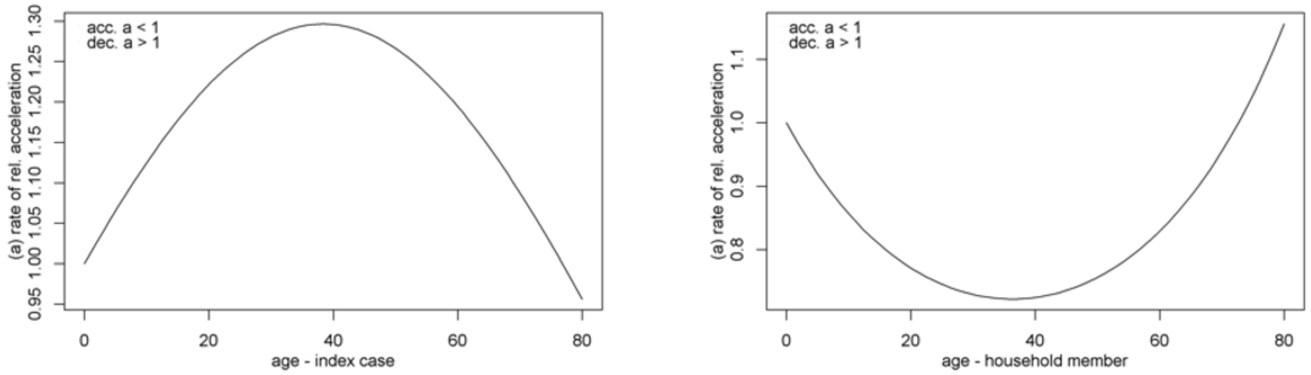


*Supplementary Figure 3: effect of age of primary case and contact on serial interval*

Age modelled as a continuous variable. a<1 reduces the serial interval a>1 prolongs the serial interval (fitting Accelerated Failure Time Model (AFT))

$Y = \log(T) = -\Sigma\, \beta X + \sigma W,\ T \sim Weibull$, *W error term ~ extreme value distribution*

$a = \exp(\,\Sigma\, \beta X)$, *factor of relative acceleration of survival function S(t,X) =* $S_0(t \exp(\,\Sigma\, \beta X))$

*Citation: https://data.princeton.edu/pop509/ParametricSurvival.pdf*

*Supplementary Table 5: Adjusted Serial Interval (for households with primary case being in the household at the time of onset), using marginal means*

| age_interaction | SI | SI_lb | SI_ub |
|---|---|---|---|
| <18 -> <18 | 3.89 | 0.83 | 6.88 |
| <18 -> 18-34 | 4.04 | 0.87 | 7.14 |
| <18 -> 35-64 | 3.39 | 0.73 | 5.99 |
| <18 -> 65+ | 4.26 | 0.91 | 7.53 |
| 18-64 -> <18 | 5.14 | 1.1 | 9.09 |
| 18-64 -> 18-34 | 5.34 | 1.14 | 9.44 |
| 18-64 -> 35-64 | 4.48 | 0.96 | 7.92 |
| 18-64 -> 65+ | 5.63 | 1.21 | 9.96 |
| 65+ -> <18 | 6.35 | 1.36 | 11.23 |
| 65+ -> 18-34 | 6.59 | 1.41 | 11.67 |
| 65+ -> 35-64 | 5.53 | 1.19 | 9.79 |
| 65+ -> 65+ | 6.95 | 1.49 | 12.3 |

Weibull fit AFT multivariable regression

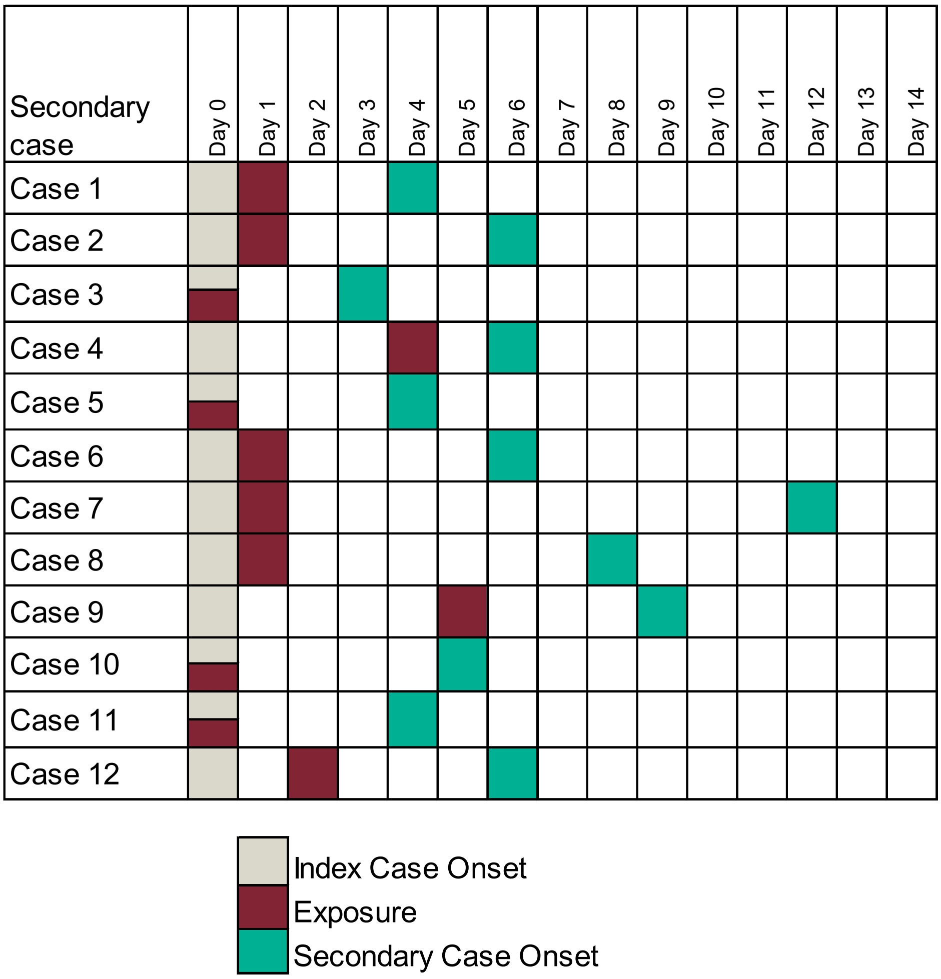


*Supplementary Figure 4: Incubation period among confirmed secondary cases with a point source exposure*
